# Glucometabolic reprogramming: From trigger to therapeutic target in hepatocellular carcinoma

**DOI:** 10.3389/fonc.2022.953668

**Published:** 2022-07-15

**Authors:** Haoming Xia, Ziyue Huang, Zhensheng Wang, Shuqiang Liu, Xudong Zhao, Junqi You, Yi Xu, Judy Wai Ping Yam, Yunfu Cui

**Affiliations:** ^1^ Department of Hepatopancreatobiliary Surgery, Second Affiliated Hospital of Harbin Medical University, Harbin, China; ^2^ The Key Laboratory of Myocardial Ischemia, Harbin Medical University, Ministry of Education, Harbin, China; ^3^ Department of Pathology, Li Ka Shing Faculty of Medicine, The University of Hong Kong, Hong Kong, Hong Kong SAR, China

**Keywords:** hepatocellular carcinoma, glucometabolic reprogramming, tumor microenvironment, drug resistance, antitumor agents, environmental stress

## Abstract

Glucose, the central macronutrient, releases energy as ATP through carbon bond oxidation and supports various physiological functions of living organisms. Hepatocarcinogenesis relies on the bioenergetic advantage conferred by glucometabolic reprogramming. The exploitation of reformed metabolism induces a uniquely inert environment conducive to survival and renders the hepatocellular carcinoma (HCC) cells the extraordinary ability to thrive even in the nutrient-poor tumor microenvironment. The rewired metabolism also confers a defensive barrier which protects the HCC cells from environmental stress and immune surveillance. Additionally, targeted interventions against key players of HCC metabolic and signaling pathways provide promising prospects for tumor therapy. The active search for novel drugs based on innovative mutation targets is warranted in the future for effectively treating advanced HCC and the preoperative downstage. This article aims to review the regulatory mechanisms and therapeutic value of glucometabolic reprogramming on the disease progression of HCC, to gain insights into basic and clinical research.

## Introduction

Hepatocellular carcinoma (HCC) is globally the most common and primary form of liver cancer, ranking fourth in cancer-related deaths (1). Radical surgical resection is the most effective way to ensure highest survival time of HCC patients (5-year survival rate> 60%), however strict surgical restrictions are difficult for most patients to meet. Sorafenib has been the standard of care and the only systemic treatment for advanced HCC for more than 10 years. In 2018, lenvatinib was approved as a first-line alternative, but did not improve the median overall survival (OS) compared to sorafenib. The combination of atezolizumab and bevacizumab was the first to show clinically meaningful improvements in all efficacy outcomes compared to sorafenib. According to the randomized phase III IMbrave150 study, the OS was 19.2 months with atezolizumab and bevacizumab versus 13.4 months with sorafenib (2). Although the current systemic treatment strategies for HCC have been improved, there is still a huge gap in the OS of patients compared with those undergone radical resection. Novel treatment strategies are still urgently needed to further improve the OS of patients with advanced HCC.

HCC cells rewire numerous metabolic pathways to achieve diverse physiological and metabolic advantages. The reprogrammed metabolism not only allows the cancer cells to sustain their uncontrolled proliferation, but also helps in creating an immune inert tumor microenvironment (TME) by interaction with non-malignant cells and extracellular matrix (3, 4). Nutrients required for the survival of organism are mainly divided into two categories: macronutrients and micronutrients. Macronutrients can be regarded as the main components of different tissues, and they are also the main energy source of the human body, represented by carbohydrates, proteins and lipids. In contrast, micronutrients are components that do not contribute significantly to caloric intake, but are still essential for important physiological functions, including vitamins and minerals (5). Glucose, the central macronutrient, releases energy as ATP through carbon bond oxidation and supports various physiological functions of living organisms. Tumor tissues prefer the metabolic mode of converting pyruvate to lactate, even under aerobic conditions, leading to a tenfold increase in glucose consumption that of nontumor tissues. This form of metabolism is called the “Warburg effect” (6), and is recognized as one of the defining hallmarks of cancer (7). Besides glycolysis, the pentose phosphate pathway (PPP) and the TCA cycle together constitute the network of central carbon metabolism, which modulates the alternative energy distribution to meet the biosynthetic requirements of HCC cells. We discussed how this unique metabolic mode promoted the aggressiveness and resistance of HCC cells, and provided promising therapeutic opportunities for novel and personalized treatments in the light of altered metabolic pathways.

### Alternative of metabolic pattern

**Figure 1:**
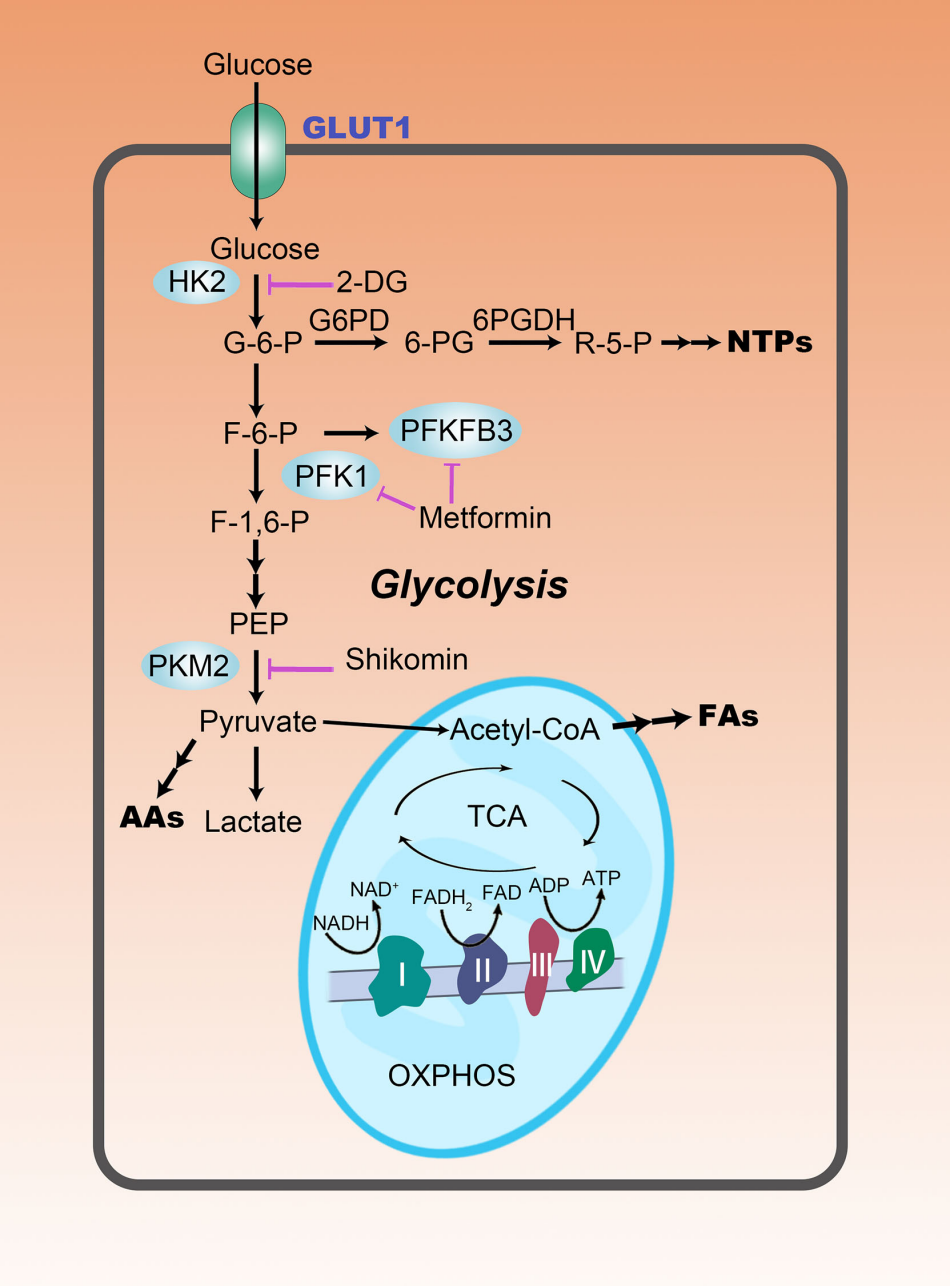
The rewiring of glucose metabolism in HCC cells. Glycolysis is abnormally active in HCC cells, even in a well-oxygenated environment. This reprogrammed metabolic pattern is manifested not only by increased glucose uptake, but also by increased lactate production. Enhanced aerobic glycolysis provides more carbon intermediates for the biosynthesis of nucleotides (NTPs), amino acids (AAs), and fatty acids (FAs), promoting anabolism in HCC cells. Purple lines represent metabolism-targeted drugs in clinical studies.

To meet the high energy and biosynthetic demands for rapid proliferation, HCC cells switch their energy metabolism. Contrary to the nontumor cells which rely mainly on mitochondrial oxidative phosphorylation (OXPHOS) for energy production, HCC cells consume an excessive amount of glucose and glycolytically convert it to lactate to obtain energy, even under aerobic conditions (**Figure 1**) (8). Sufficient glycolysis flux seems to have formed an inefficient production model, but its shorter time-consuming to some extent compensates for the efficiency shortcomings. Furthermore, aerobic glycolysis provides several other unmatched metabolic advantages, such as generation of metabolic intermediates and substrates for biosynthesis, maintenance of redox balance and providing conducive immunosuppressive microenvironment (9), for HCC cells.

#### Aerobic glycolysis

The rewiring of glucose metabolism is accompanied by altered expression of three rate-limiting enzymes of glycolysis. (1) HK2, an isozyme of Hexokinase (HK), catalyzes the conversion of glucose into glucose-6-phosphate (G6P) and is overexpressed in HCC tissues. HK2 activates the voltage-dependent anion-selective channel protein 1 (VDAC1), promotes glycolysis and triggers rapid ATP production (10). (2) Phosphofructokinase 1 (PFK1) catalyzes the phosphorylation of fructose 6-phosphate (F6P) to produce fructose-1,6-bisphosphate (F-1,6-BP) (11). PFK1 activity increases in HCC. Dimeric or tetrameric PFK1 is rapidly formed from the unstable yet active monomers, thus, increasing the glycolytic flux (12). (3) Pyruvate kinase (PK) catalyzes the transfer of phosphate from phosphoenolpyruvate (PEP) to ADP producing pyruvate and ATP. PKM2, one of the isoforms of PK, forms tetramers in the cytoplasm to increase the catalytic efficiency (13), or transfers to the nucleus in its dimeric form to activate the transcription factors associated with tumorigenesis (14). Pyruvate is subsequently catalyzed by lactate dehydrogenase (LDH) to lactate and yields NAD+ from NADH and H+ (15). As the main isoenzyme in HCC, LDHA drives the rapid conversion of pyruvate to lactate, enhancing the Warburg effect and the flux of glycolysis, which is essential for keeping the glycolytic pathway activated (7). To manage the excessive intracellular acidic metabolites generated due to rapid aerobic glycolysis, the expression of monocarboxylate transporter (MCT) is elevated. This transporter on the plasma membrane of HCC cells accelerates the transport of lactate to the extracellular microenvironment (15). CD147, a transmembrane glycoprotein, can induce the expression of matrix metalloprotease (MMP), which along with MCT4, synergistically facilitates the secretion of lactate (16). Detailedly, each domain of CD147 can interact with different proteins and exhibit different functions. N-glycosylation of the asparagine residue at the 44th position of the ECI domain is critical for its MMP-inducing activity and homotypic interactions (17). The interaction of the ECII domain with caveolin-1 has also been shown to regulate CD147 aggregation at the plasma membrane and modulate its MMP-inducing activity. Furthermore, the transmembrane domain of CD147 can interact with proteins such as MCT-1 and MCT-4, which may be attributed to the interaction of the glutamate residue of CD147 with the arginine residue of MCT (18).

#### Glycolysis related signaling alternations

The rapid progression of HCC is often accompanied by a severe imbalance in oxygen levels at the center of the tumor. The tumor cells at the core are challenged with oxygen deprivation and hence must adapt to the environmental challenge of hypoxia by activating the hypoxia-inducible factor-1 α (HIF-1α) transcription factor (19). By binding to the hypoxia responsive elements (HRE) of the target gene promoter, HIF-1α induces the overexpression of glycolytic enzymes, GLUTs, and pyruvate dehydrogenase kinase (PDK). This steers the metabolic switch from OXPHOS to glycolysis (20). HIF-1α also mediates vascular endothelial growth factor (VEGF)-stimulated formation of new blood vessels in hypoxic conditions (21). Additionally, HIF-1α can also stimulate the metastasis of HCC to more oxygenated tissues by activating carcinogenic growth factors such as transforming growth factor beta3 (TGF-β3) and epidermal growth factor (EGF), etc. (22). Besides, there are other signaling pathways involved in the metabolic regulation in HCC. For instance, phosphatidylinositol 3-kinase (PI3K)/Akt signaling, a convergence point of receptor tyrosine kinases and extracellular matrix signals, can act as a crucial regulator of glucose uptake. It promotes the expression of glucose transporter 1 (GLUT1) and its transport from the inner membrane to the cell surface (23). By interacting with AMP-activated protein kinase (AMPK) and HIF-1α, PI3K/Akt indirectly regulates the expression of glycolytic enzymes (24), and further activates HIF-1α by stimulating the downstream regulators of the mammalian target of rapamycin (mTOR), which favors aerobic glycolysis and angiogenesis (25). c-Myc has been confirmed to be involved in the occurrence and development of various tumors. The oncogenes KRAS and ERK can partially increase the levels of c-Myc by alleviating its stability (26). c-Myc activates the expression of key enzymes of glycolysis and favors the expression of specific mRNA splice variants, such as PKM2 over PKM1 (27). The accumulation of glycolysis intermediates driven by c-Myc has long-standing effects on other metabolic pathways. Due to the c-Myc-driven glucose shunt, PPP uses glycolysis-derived G6P as the starting substrate to produce the reduction equivalent of NADPH and the central molecule of nucleotide biosynthetic pathway, ribose (28). Additionally, c-Myc-regulated, serine hydroxymethyltransferase-2 (SHMT2)-dependent NADPH production is considered necessary for the maintenance of redox balance and survival of myc-transformed cells under hypoxic conditions (29).

#### Mitochondrial dysfunction and dynamic network

**Figure 2:**
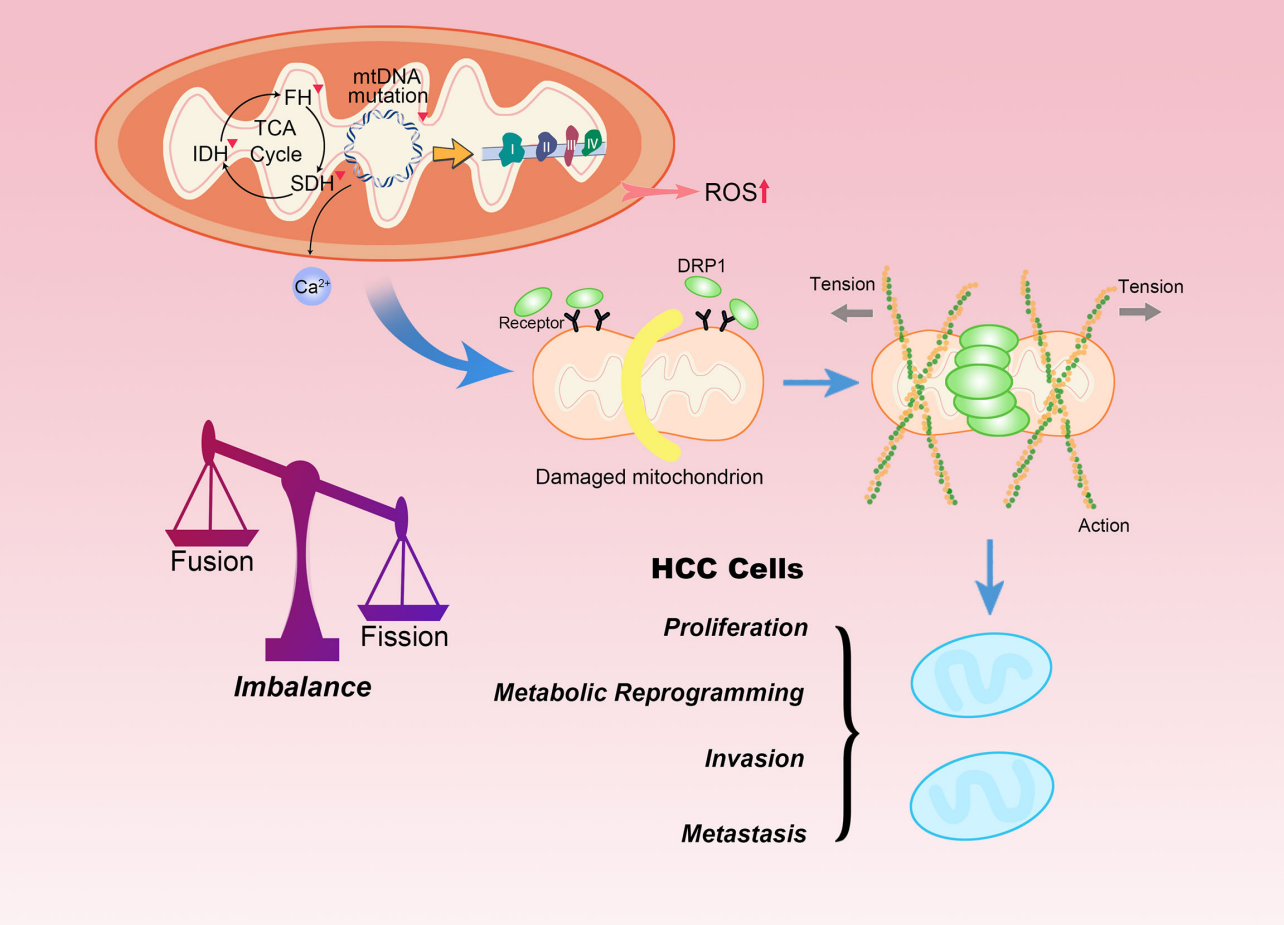
Mitochondrial dysfunction and fission promote HCC progression. Mutations in mtDNA and defects in mitochondrial enzymes, such as SDH, FH and IDH are closely related to familial and sporadic cancers. The accumulation of ROS, Ca^2+^ or carcinogenic metabolites can participate in tumor transformation. Moreover, mitochondrial morphology is interchangeable between fused and fission states. Mitochondrial fission begins with interaction with endoplasmic reticulum. DRP1 is recruited to mitochondria upon stress, where it interacts with its mitochondrial receptors. DRP1 is responsible for mitochondrial fission as it physically confines mitochondria by forming a ring structure in the region of future mitochondrial fission. The level of mitochondrial fission significantly affects the malignant biological behaviors of HCC.

The lack of histone proteins, the protection rendered by them, and effective DNA repair mechanisms make mitochondrial DNA (mtDNA) extremely vulnerable to DNA damage, which contributes to the dysregulation of the OXPHOS system, enhanced ROS generation and Ca2+ mobilization, leading to further occurrences of mutations in DNA (**Figure 2**) (30). Mutations and copy number reduction of mtDNA may contribute to mitochondrial dysfunction, and its pro-oncogenic effect can be attributed to the functional attenuation of some gene products. Mitochondrial gene inactivation and dysfunction result in the increase of ROS levels in the cytoplasm as well as in mitochondria caused due to the escape of electrons from the respiratory chain (31). Most importantly, mitochondrial dysfunction renders a multi-faceted effect on various pathways, including the integrin pathway, the platelet-derived growth factor (PDGF) signaling pathway, and the cadherin signaling pathway. Altered signal transduction in all these pathways stimulates metastasis of the HCC to a certain extent (32). However, mtDNA depletion is sometimes compensated by upregulating manganese superoxide dismutase (MnSOD) and other antioxidant enzymes to restore the metabolic adaptability of cells in oxidative stress conditions (33).

Mitochondria exist in the form of a dynamic network, whose remodeling is mechanically regulated by key dynamin-related fission and fusion gene products, responsive to changes in energy requirements or stress (34). HCC cells with high metastatic potential lower the expression of mitochondrial fusion protein mitofusin-1 (MFN1), steering the mitochondrial dynamics specifically toward fission. This disruption in the dynamic balance between fission and fusion leads to more fragmented mitochondria. The lack of functional mitochondria prompts the transformation of the metabolic phenotype of HCC cells from OXPHOS to aerobic glycolysis (35). On the contrary, the fusion of mitochondria can promote respiration by enhancing the dependence of Opa-1 on cristae density and the formation of respiratory chain supercomplexes. Additionally, fusion also accelerates the uptake of pyruvate or other OXPHOS substrates (36). Interestingly, some proteins that are not originally mitochondrial proteins or transported to mitochondria under unperturbed physiological conditions can be transferred to mitochondria under pathological conditions. This phenomenon of aberrant mitochondrial transport is known as mitochondrial translocation, which is another feature commonly observed in HCC. Phosphorylation in Ser34 position of the macroautophagy/autophagy-related protein autophagy related 4B cysteine peptidase (ATG4B) can promote its own enrichment in mitochondria in HCC. The translocated ATG4B interacts with the ATP5C1-containing complex in the mitochondria and inhibits F1Fo-ATP synthase activity. Thus, inhibition of ETC suppresses the mitochondrial function and promotes the Warburg effect, with a minor effect on autophagy flux (37). PDSS2 is downregulated in HCC. This diminishes the concentration of CoQ10 and weakens the transport of electrons and the antioxidant defense mechanism in the mitochondrial respiratory chain. Thus, the oxidative respiration ability of cells is abolished by downregulating the key enzymes of the TCA cycle (38). Unlike non-cancer stem cells (CSCs), the maintenance of stemness of hepatic CSCs largely depends on the enhanced mitochondrial function induced by mitochondrial ribosomal protein S5 (MRPS5). The increased activity of mitochondria is characterized by the production of a large number of NAD+, NAD+ precursors and ROS. The elevated levels of NAD+ dependent deacetylase, sirtuin-1 (SIRT1) in hepatic CSCs maintains the deacetylation of MRPS5. The mitochondrial function is strengthened by the translocation of MRPS5 from the nucleus to the mitochondria (39).

### Linking glucometabolic reprogramming to stress responses

#### Oxidative and energy stress

Glycolysis-mediated increased glucose directly or indirectly eventually drives the flux to PPP to produce NADPH (40), which is further required for the conversion of oxidized glutathione (GSSG) to reduced GSH (41). This conversion restores a conducive reducing environment to resist oxidative stress. The increased activity of the overexpressed TKT enzyme (the key player in the non-oxidative phase of PPP) in HCC contributes to the production of NADPH, producing the reducing power and maintaining the balance between NADPH/NADP and GSH/GSSG ratios (42). However, during the rapid growth process, HCC cells often face the disparity between the high glucose demand for rapid proliferation and the deficient supply. Under metabolic stress, the production of NADPH from glycolysis is suppressed due to the reduced utilization of PPP (43). Additionally, glucose unavailability leads to excessive burden for energy on mitochondria, restoring the metabolism from glycolysis to OXPHOS, and the subsequent production of pro-oxidants causes ROS overload (44). The imbalance between the production and elimination of ROS causes oxidative stress, leading to varied deleterious outcomes including, disruption of cellular structural integrity, DNA mutation and damage to genome integrity, eventually leading to cell senescence and death (45). This environment prompts HCC cells to utilize a compensatory mechanism to neutralize the energy and oxidative stress in the TME. Overexpression of O-GlcNAc transferase (OGT) in HCC can combat hyperactive mtOXPHOS by triggering aberrant O-GlcNAcylation of Rab3A in HCC (46). MRPS5 and NAD+/SIRT1 signaling are involved in maintaining the NAD+/NADH ratio in hepatic CSCs, reducing punctate mitochondria and disorganized cristae, and inducing the mitochondrial unfolded protein response (UPRmt). All these compensatory measures are taken to recover from the oxidative damage in hepatic CSCs due to enhanced oxidative respiration (39). Moreover, the death of HCC cells triggered by depletion in carbon source causes the cells to release thymidine and other remaining carbon sources. Thymidine phosphorylase (TP) catalyzes the extracellular metabolism of thymidine to ATP and amino acids *via* the pentose-Warburg effect. This provides energy for the remaining surviving cells (47).

#### Autophagy

**Figure 3:**
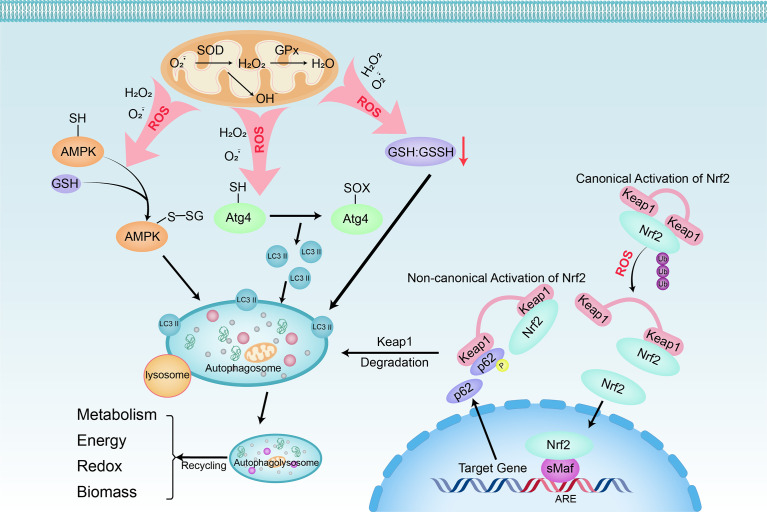
Linking oxidative stress to autophagy in HCC cells. Oxidative stress can be triggered by any dangerous or inflammatory signal and affects many cells in the liver. Superoxide (O2−) and H2O2 are the main ROS produced by mitochondria during nutrient deprivation. Three main regulatory pathways are involved the positive regulation of autophagy by ROS: 1) S-glutathionylation of cysteine located in the α and β subunits of AMPK (SH → S-SG); 2) The oxidation of Cys81 (SH → Sox) of Atg4 leads to the accumulation of LC3 II; 3) decrease of GSH : GSSH ratio. Furthermore, with oxidative and electrophilic stress, the oxidation of cysteine residues regulated by Keap1 will inhibit the ubiquitination of Keap1 and Nrf2, resulting in canonical Nrf2 activation and induction of target gene expression. When p62 is phosphorylated at Ser349, it binds to Keap1, causing its autophagy degradation. After that, Nrf2 freely transfers to the nucleus and induces target gene expression (non-canonical Nrf2 activation). When autophagosomes fuse with lysosomes that provide hydrolytic enzymes, the cargo is degraded. The breakdown products of autophagy are released into the cytoplasm, where they are recycled into metabolic and biosynthetic pathways.

Autophagy is a catabolic process of degradation mediated by lysosomes. In this process, cells recycle intracellular macromolecules and organelles to regenerate building blocks. Stimulation of autophagy facilitates HCC cells’ survival under stressful metabolic and environmental conditions and combats the potentially deleterious effects of mitochondrial dysfunction and ROS generated by organelles (**Figure 3**). Because catabolism is in contrary to the rapid mass accumulation required for hepatocarcinogenesis, inhibition of autophagy driven by oncogenes such as Ras, PI3K, and myc is often an early event (48). PI3K/AKT/mTOR, an important regulatory pathway upstream of autophagy, also plays a key regulatory role in hepatocarcinogenesis. Activated AKT and PI3K in HCC cells contribute to the activation of mTORC1. When active, the downstream effectors of mTORC1, S6K and 4E-BP, are phosphorylated to promote anabolic processes such as protein, lipid, and nucleotide synthesis, thereby inducing cell growth and proliferation. Simultaneously it inhibits the catabolic program through unc-51-like autophagy activating kinase 1 (ULK1), thereby inhibiting autophagy (49, 50). Moreover, DNA damage and chromosomal instability due to defects in autophagy also render cells with unique tumorigenic properties (51). The phosphorylated autophagy chaperone proteins p62 and Keap1 accumulate in tumors deficient in autophagy genes (Atg5, Atg7) and form aggregates, leading to the continued activation of Nrf2 (52). On one hand, the transcription factor Nrf2 can mediate the phosphorylation of p62 at Ser349 to drive glucose to the glucuronic acid pathway, non-oxidative PPP, and purine nucleotide synthesis, and steer glutamine to glutathione synthesis, thereby conferring HCC the tolerance against anticancer drugs, sorafenib and cisplatin (53). On the other hand, Nrf2 along with atypical protein kinase Cs PKCλ/ι can oppose PPAR-α-dependent OXPHOS by inhibiting the expression of PKCζ and minor phosphorylation of LC3 to further prevent hepatocyte autophagy (54).

The above studies corroborates the tumor suppressor effect of autophagy during the development of HCC. However, assessing the complete time course of tumor formation, it seems that autophagy may also protect cancer cells from the metabolic pressure inflicted by chemotherapy and the environment (55). For instance, hepatocyte growth factor (HGF) and its receptor tyrosine kinase MET were first discovered in the 1980s due to their over-activation in liver cancers. HGF-MET axis can accelerate the glucose-to-lactate and glutamine-to-glutamate transformation and effectively induce HCC. Autophagy is highly active in cells resistant to drugs targeting the HGF-MET kinase. Under the conditions where kinase is inhibited, Y1234/1235 dephosphorylated MET drives mTOR-dependent autophagy for cancer biogenesis (56). Another potent drug, CP-91149, an allosteric inhibitor of glycogen phosphorylase (GP), limits the availability of glucose derived from cellular glycogen, mediates the nutrient starvation state of cells, and accompanies autophagy. Autophagy adaptively neutralizes the effect of changes in glucose and pyruvate levels on cells, reducing the drug efficacy of CP-91149 (57). Since markers of increased autophagic activity often highly expressed in tumor tissues, previous studies have established that autophagy may be repressed at the initial stage of transformation, but is upregulated after the tumor is fully formed (58). However, research to date has not yet elucidated the transformation node of the inhibitory/promoting effect between autophagy and liver cancer and the specific underlying molecular mechanism of the transformation. The treatment strategy aims at limiting the progression of HCC by increasing or reducing the autophagy flux of the patient’s tumor tissue. However, further research is warranted to unravel how to meticulously balance the promotion and inhibition of autophagy to effectively treat HCC.

### HBV-induced metabolic switch

Changes in the metabolic network in HBV-infected liver cells activate many downstream carcinogenic pathways that contribute to the cancerous transformation of cells (59). The key enzymes of glycolysis pathways (HK2, ALDOA, PKM2, etc.) are significantly upregulated in HBV-associated HCC, which indicates an increased glucose metabolism. HBV core protein (HBc) activates the expression of aldolase, fructose-bisphosphate C (ALDOC) and phosphoenolpyruvate carboxykinase 1 (PCK1) by recruiting the transcription factor MAX-like protein (MLX) (60). Additionally, in catenin beta 1 (CTNNB1) mutant HCC, the increased phosphorylation of ALDOA at Ser36 can also promote glycolysis and subsequently facilitate tumor growth (61). Chronic HBV infection is also accompanied by a metabolic mode of CD8+ T cells centered on mitochondrial dysfunction and overwhelming ROS production (62), which makes it difficult for T cells to drive the metabolic reprogramming from glycolysis to OXPHOS and meet the biosynthetic needs of T cells (63).

The insertion of viral DNA into the host genome does not contribute to HBV replication but is an important step in viral pathogenesis both due to the cis-acting mechanisms and the constitutive expression of trans-acting wild-type and truncated hepatitis B virus x protein (HBx) (64). On one hand, HBx can induce the formation of HBx-p62-Kelch-like ECH-associated protein 1 (Keap1) complex in the cytoplasm, promoting the PPP pathway by activating nuclear factor erythroid 2–related factor 2 (Nrf2) and subsequent transcription upregulation of G6PD (65); On the other hand, HBx can inhibit the expression of Glycogen synthase 2 (GYS2), an inhibitor of MDM2-mediated ubiquitination and degradation of p53, in HBV-related HCC by forming a HBx/histone deacetylase 1 (HDAC1) complex to promote the progress of HCC, exhibiting an antagonistic effect on glycogen synthesis (66). The breakpoints at the 3’-end of the HBx gene occur in tumor-specific events that lead to the production of truncated Ct-HBx protein (67). Ct-HBx accelerates glucose uptake through the interaction of transcriptional repressor nuclear factor of activated T cells 2 (NFACT2) and the negative regulator of glucose uptake, thioredoxin interacting protein (TXNIP). This interaction activates HIF-1α through the p-mTOR, inducing the overexpression of the regulators of the glycolysis cascade (68). The oncoprotein Hepatitis B virus X-interacting protein (HBXIP) can positively regulate methyltransferase-like 3 (METTL3), an RNA N6-adenosine methyltransferase. METTL3 catalyzes the methylation of adenosine forming N6-methyladenosine (m6A) thereby inhibiting aerobic glycolysis and OXPHOS of HCC cells. This promotes the malignant behaviors of tumor cells (69). Additionally, the upregulation of ATP-binding cassette sub-family G member 2 (ABCG2)-dependent stem-like side population (SP) cells, regarded as liver tumor stem cells, compared to the HBx-expressing HCC cells suggests the plausible participation of HBx in acquiring the stemness in HCC cells. BNIP3-like (BNIP3L) signaling is one of mitophagy regulatory pathways. BNIP3L located in the outer mitochondrial membrane interacts with processed LC3 to promote the occurrence of autophagy. BNIP3L-dependent mitochondrial autophagy is activated in SP cells and HBx-expressing HCC cells, steering the metabolic shift towards glycolysis. However, the intervention of glycolysis can attenuate the tumor stem-like phenotype (70). However, to elucidate the precise regulatory correlation between HBx and mitochondrial autophagy or glycolytic transformation further investigation is required.

### Crosstalk between HCC and immunosuppressive microenvironment

**Figure 4:**
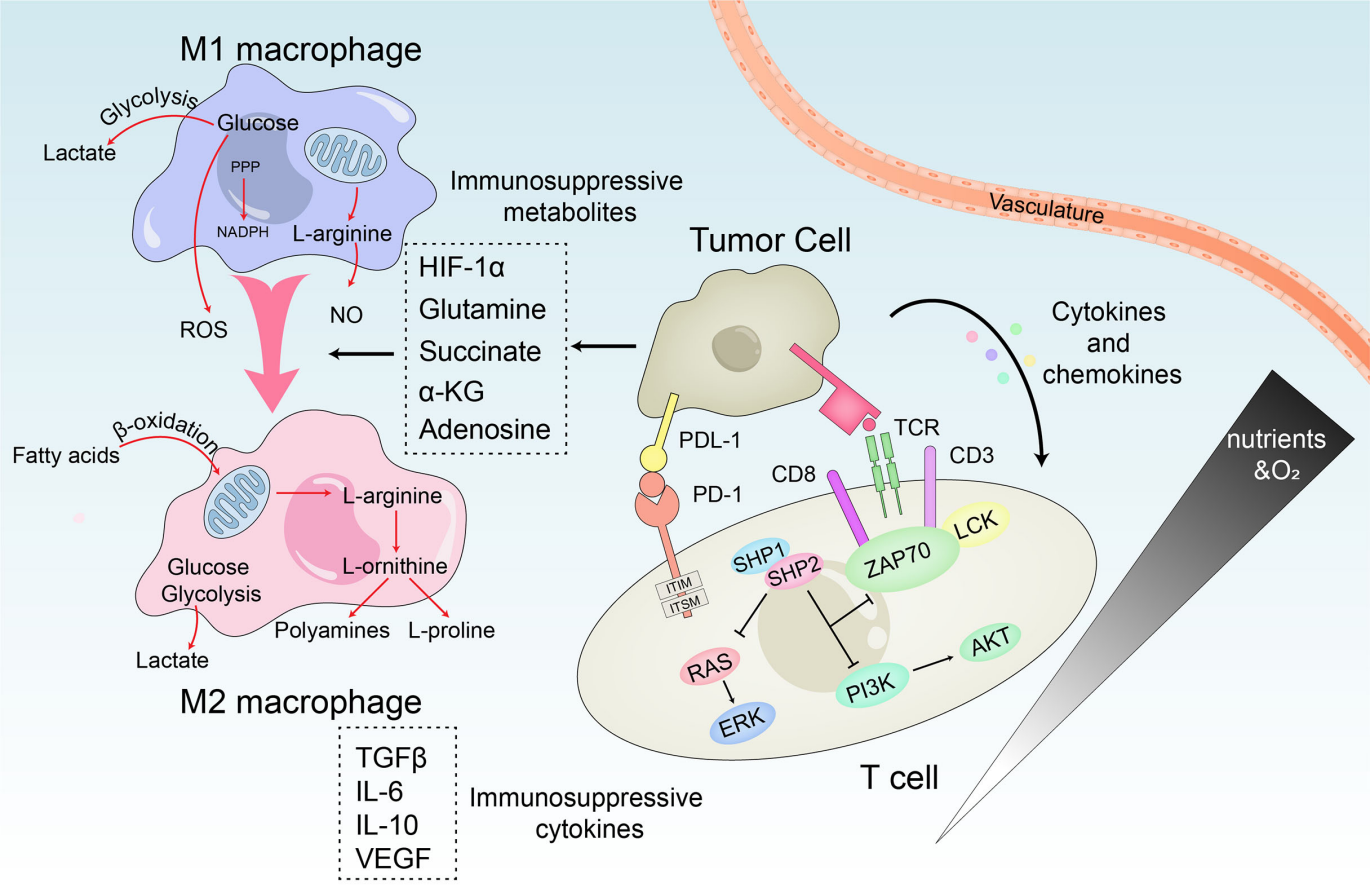
Metabolic crosstalk within the immunosuppressive microenvironment. M1 macrophages rely on the production of lactate, ROS and NO to kill tumor cells, while M2 macrophages mainly promote tumor growth through β-oxidation of fatty acids and polyamines and L-proline produced by the TCA cycle. In HCC, TME reprogram macrophage metabolism through immunosuppressive metabolites and induces the repolarization of M1 to M2. Similarly, the interaction of immune regulatory cells and cytokines disables the anti-tumor T cell response. PD-1 accompanied with PD-L1 directly inhibits T cell receptor signaling by preventing phosphorylation of ZAP70. Meanwhile, the PI3K/Akt and Ras/MEK/Erk pathways required to initiate T cell activation are also be disrupted.

The interaction between HCC cells, the infiltrating immune cells, and their secreted cytokines significantly affects the process of tumorigenesis. Surprisingly, various immune cells which are generally regarded as the principal effectors of the host defense system might also promote tumorigenesis in the inflammatory environment. The immunosuppressive microenvironment of HCC is primarily associated with T cells, natural killer (NK) cells and tumor-associated macrophages (TAMs). The dysfunction of immune cells reduces inflammation and suppresses immune response which changes the microenvironmental functions ultimately contributing to the progress of HCC (**Figure 4**).

One of the most abundant types of stromal cells in TME is tumor-infiltrating macrophages, which originate from circulating monocyte precursors. These macrophages are stimulated by tumor cell-derived regulatory factors and differentiate into mature macrophages (71). Based on different activation states, macrophages can be classified into M1 and M2 subtypes. TAMs are M2-like macrophages (72). TAMs are typically characterized by their production of anti-inflammatory factors (IL-6, IL-10, TGF-β) and ability to induce angiogenesis, immune escape, causing tumorigenesis (73). Mediators of signaling derived from tumors or immune cells induce the immune imbalance of the TME, in a way that the macrophages lose their innate anti-tumor activity, while enhancing their tumor-promoting activity. The conversion of pyruvate to lactate by aerobic glycolysis and the accumulation of lactate lowers the pH to 6.0-6.5 leading to acidosis in HCC (74). On one hand, the accumulated lactate induces the expression of VEGF mediated by HIF-1α, and activates the NF-κB and PI3K signals of endothelial cells to promote angiogenesis (75). On the other hand, it promotes the polarization of M2-like macrophages by activating G protein-coupled receptor 132 (Gpr132) (76), deactivates dendritic cells (DC) and T cells, and prevents the migration of monocytes to participate in the formation of the immune microenvironment (77). In addition, the upregulated glycolytic regulator 6-phosphofructo-2-kinase/fructose-2,6-biphosphatase 3 (PFKFB3) in TAMs is associated with the expression of programmed cell death ligand 1 (PD-L1) to promote immune escape and recurrence of HCC (78). HCC-derived ectosomal PKM2 induces glycolytic reprogramming in monocytes, by mediating the phosphorylation of signal transducer and activator of transcription 3 (STAT3) in the nucleus. Thereafter, STAT3 can be recruited to the promoters of the related transcription factors (MafB, c-Maf, Egr-1, and Klf4) to stimulate their overexpression, leading to the differentiation of monocytes into M2-like macrophages instead of DCs. On the other hand, glycolysis can increase the concentration of acetyl-coenzyme A (CoA) thereby promoting the acetylation of histone H3K9 and H4K8 in monocytes. This leads to increased levels of H3K9 and H4K8 acetylated by the pro-differentiation TFs promoter, thereby driving monocytes to differentiate into M2-like macrophages and remodel the TME. Sulfonylated PKM2 interacts with arrestin-domain-containing protein 1 (ARRDC1) to induce its transport to plasma membrane surface and subsequent ectosomal excretion. This contributes to the ectosomal secretion of PKM2 from HCC cells. In addition, the cytokines/chemokines secreted by macrophages enhance the association of PKM2-ARRDC1 in a CCL1-CCR8 axis-dependent manner. This further induces the secretion of PKM2 from HCC cells creating a positive feedback regulatory loop promoting tumorigenesis (79).

Differential regulation of transcription drives the differentiation of activated T cells into effector T cells and memory T cells, with entirely different metabolic characteristics. Mitochondria are not only essential for survival of T cells but also for its activation. Partly through the generation of ROS, mitochondria facilitates the activation of nuclear factor of activated T cells (NFAT) and the production of IL-2, which is an important factor in T cell function. The T-cell receptor (TCR) signal in effector T cells promotes mitochondrial fission which leads to lower respiratory activity. On the contrary, the increased number of mitochondria in memory T cells and the fused ultrastructure confer enhanced respiratory capacity, which supports cell survival in the absence of antigen (80, 81). However, continuous antigen stimulation such as HCC or chronic viral infections induces T cells to exhibit a unique exhaustion phenotype where they gradually lose their proliferation potential and productivity of effector cytokines. The expression of programmed cell death protein-1 (PD-1) and Tim-3 are suppressed and while that of the Lag-3 receptors gradually increase (82, 83). The immunomodulatory cells and HCC cells in TME can exert metabolic stress on the tumor-infiltrating lymphocytes (TILs). They can also coordinate to impair the anti-tumor response of T cells, and induce T cell exhaustion in various ways. 1) Firstly, CD8+ TILs induce mitochondrial depolarization due to the decreased expression of transcriptional co-activator peroxlsome proliferator-activated receptor-γ coactlvator-1α (PGC-1α), leading to the substantial production of mtROS and the terminal failure characteristics of TILs (84). 2) Also, the lack of CD4+ T cell nuclear immunosuppressive factors, the inhibitory effect regulating T cells (Treg cells) and the continued inhibitory receptor signals can also result in T cell failure (85). 3) Additionally, the increased accumulation of TAMs can also lead to T cell failure due to the production of IL-10 and the expression of PD-L1, which weakens their anti-tumor response (86). Immune checkpoints can also become negative regulators of immune response by inhibiting effector T cells. PD-1 and cytolytic T lymphocyte-associated antigen-4 (CTLA-4) are the most abundant checkpoints of T cells, which are intricately correlated with glucose metabolism (87). Moreover, elevated levels of PD-1 are characteristic of exhausted T cells (88). HCC cells expressing myocyte specific enhancer factor 2D (MEF2D) can upregulate their PD-L1 expression which increases their binding to PD-1 (89). PD-1 can induce metabolic reprogramming of T cells by inhibiting the expression of principal glucose transporter GLUT1 and the key glycolytic enzyme HK2. At the same time, it also promotes mitochondrial dysfunction, ultimately leading to shift in the metabolism of HCC.

Unlike CD8+ T cells, NK cells can quickly and spontaneously recognize and kill viral infections as well as HCC cells, independent of specific neoantigen recognition (90). NK cells release cytotoxic granules containing cytolytic enzymes perforin and granzymes to lyse target cells. Among the two human NK cell subgroups CD56dim and CD56bright, CD56dim exhibits stronger cytotoxicity, while CD56bright is superior cytokine producer and exhibits stronger metabolic activity (91). Glycolysis is necessary for NK cells to maintain the production of IFN-γ and granzyme B and is regulated by mTOR (92). The rapid proliferation of HCC cells results in the lack of glucose, glutamine, and oxygen in the TME, while end-product metabolites such as lactate accumulate in large quantities (93). NK cells are adversely affected by glucose depletion. TGF-β causes aberrant F-1,6-BP expression, which inhibits glycolysis and leads to NK cell dysfunction by impairing its viability (94). In addition, the inhibitory TME metabolite adenosine produced by the hypoxia-driven exoenzyme CD73 downregulates the expression of genes involved in metabolism. This significantly impairs the glycolytic and other metabolic functions of NK cells (95). Hypoxia-induced mitochondrial fission results in the increase of small and fragmented mitochondria in HCC cells. This inhibits the viability of NK cells and impairs their cytotoxic effects against HCC cells to a certain extent, which enables HCC to evade the immune response (96). Hypoxic tumor-resident NK cells can also upregulate VEGFR-1 by overexpressing HIF-1α, thereby inhibiting non-productive VEGF-driven angiogenesis, and promoting HCC growth by generating functional blood vessels (97). Moreover, lactate can reduce the cytotoxicity of NK cells and inhibit the upregulation of NFACT (98, 99).

### Therapeutic strategies targeting rewired metabolism

#### Targeting aerobic glycolysis

The reprogrammed glucose metabolism cascade not only ensures a steady supply of energy for the HCC cells, but also promotes the functions of many cancer proteins. At the same time, oncogenic signal molecules such as Akt can also help reprogram the progress of glucose metabolism by increasing the expression of glucose transporters on the cell surface and regulating the enzyme activity in the glucose metabolism cascade. This bi-directional interplay highlights the important role of glucose metabolism in hepatocarcinogenesis and makes targeted metabolic changes an attractive treatment strategy for HCC.

Canagliflozin (CANA) is a new energy-dependent sodium/glucose cotransporter 2 (SGLT2) inhibitor that can block SGLT2-mediated glucose uptake. The dual inhibitory function of CANA against β-catenin signaling leads to a significantly improved survival rate of HCC-bearing mice (100). Diabetes is an independent risk factor for the development of HCC in patients with nonalcoholic steatohepatitis (NASH). Dipeptidyl peptidase-4 inhibitor (DPP4i), an antidiabetic agent, can significantly inhibit the volume and number of *in situ* tumors in NASH-associated HCC mouse models. DPP4i significantly improves the hepatic steatosis and increases the levels of 6-phosphogluconic acid and ribose 5-phosphate. It also downregulates PPP by inhibiting the p62/Keap1/Nrf2 pathway (101). Another drug, Shikonin suppresses glycolysis by specifically inhibiting PKM2. The combination of doxorubicin and Shikonin are synergistic at a higher level of cytotoxicity, whereas the combination of Shikonin with cisplatin has an additive effect. Adding PKM2 inhibitors to the Transarterial Chemoembolization (TACE) procedure can overcome drug resistance and optimize the therapeutic effect of TACE (102). As discussed earlier PI3K/Akt signaling pathway promotes cell proliferation, and PI3K/Akt/mTOR signaling pathway promotes aerobic glycolysis. In this regard, an Akt inhibitor, MK2206, can reduce glucose consumption and lactate production, and significantly inhibit the growth of HCC cells. Corroborating this, oral administration of MK2206 showed an inhibitory effect on the growth of transplanted HCC in nude mice (103). Verteporfin targets the interface between Yes-associated protein (YAP) and TEA domain transcription factor (TEAD), which prevents the formation of YAP-HIF1α. Another potent drug is the direct inhibitor of HMGB1, glycyrrhizin. Both Verteporfin and Glycyrrhizin can limit the proliferation of HCC cells and the growth of HCC tumors, by suppressing the expression of genes involved in glycolysis. Inhibition of HMGB1-YAP-HIF-1α pathway can prevent the increased glycolysis and subsequently the tumor growth in mice (104). Aminooxyacetate (AOA), an inhibitor of transaminase activity, abrogates the alanine aminotransferase glutamic-pyruvic transaminase 1 (GPT1) activity and effectively reduces the level of intracellular ATP in HCC. Therefore, AOA reverses the alanine-mediated proliferation of HCC and transforms the growth pattern of tumor cells to a non-invasive pattern. In addition, the naturally occurring alkaloid, berberine (BBR), can also inhibit the activity of GPT1 in a dose-dependent manner. The alanine-dependent metabolic reprogramming, production of ATP and the glucose-alanine cycle of HCC is abolished by BBR, which inhibits the growth of HCC without inducing cytotoxicity (105). Another potent competitive inhibitor of p300, B029-2, occupies the binding pocket of Acetyl-CoA and specifically abrogates the acetylation of H3K18 and H3K27, thereby interfering with the metabolic reprogramming of HCC cells. B029-2 retards the growth of xenografts by inhibiting the major biosynthetic pathways of proteins, amino acids, and nucleotides in HCC cells. It also substantially reduces the glycolytic capacity and reserves and exhibits negligible side effects *in vivo* and *in vitro* (106). Natural polyphenolic proanthocyanidins-B2 (OPC-B2) is another useful anti-tumor drug. OPC-B2 is composed of two epicatechins, with strong antioxidant activity due to its phenolic hydrogen atoms. This antioxidant activity can effectively intercept free radicals in free radical chain reactions. In addition, OPC-B2 can bind to the catalytic and regulatory domain of oncogenic kinase Akt and locks it in a “closed” conformation to inhibit its activity. Therefore, OPC-B2 effectively abrogates HK1-mediated glycolysis in a dose-dependent manner. It also exhibits a encouraging synergistic effect in combination with the allosteric inhibitor of Akt, MK-2206 (107).

#### Combined therapy with multikinase inhibitors

Sorafenib, a multiple-target tyrosine kinase inhibitor (TKI), can inhibit the proliferation of HCC cells by inhibiting the kinase activity of Raf-1, B-Raf, and Ras/Raf/MEK/ERK signaling pathways. However, only close to 30% of patients benefit from monotherapy with sorafenib alone, and even this population often acquires drug resistance within 6 months of treatment, thereby limiting the success rate of sorafenib. In this context, the combination of drugs used in conjunction with sorafenib might ameliorate resistance and prolong the survival of HCC patients.

Sometimes the compensatory upregulation of OXHPOS and the metabolic reprogramming associated with increased glycolysis can still support the survival of HCC cells under high-dose of sorafenib treatment (108). In this regard, metformin is used along with sorafenib to produce a synergistic therapeutic effect on liver CSCs tumor-bearing mice. Metformin has an inhibitory effect on complex I which strengthens the anti-tumor effect of sorafenib, and restricts the proliferation of liver CSCs thereby impeding tumorsphere formation (39). Another important enzyme of glucose metabolism, PDK can enhance the chemoresistance of HCC cells by repressing mitochondrial PDH Complex. PDK inhibitor dicoumarol (DIC) or dichloroacetate can decelerate the metabolic transformation of HCC cells from glycolysis to OXPHOS and improves the sensitivity of HCC cells to sorafenib or cisplatin (109, 110). HCC cells can often adapt to increased oxidative stress. Malic enzyme (ME) catalyzed reduction of NAD(P)+ to NAD(P)H can effectively prohibit the accumulation of ROS in HCC cells and favors their survival. LaCl_3_, the competitive inhibitor of MEs, exhibits potential anti-tumor effects both *in vivo* and *in vitro* by reducing the NADPH/NADP+ ratio (111). ME1 and ME3 are positively regulated by Nrf2 and super-enhancers, respectively. Nrf2 inhibitor, clobetasol propionate (CP), and super-enhancer inhibitor JQ1 both effectively increase ROS levels in a dose-dependent manner. The combination of CP or JQ1 with sorafenib efficiently reduces the mass and volume of subcutaneous HCC without causing any cytotoxicity as reflected by the unaltered body weights of mice (111). Therefore, the combined drug therapy continues to be one of the preferred treatment strategies in ameliorating HCC.

The development of HCC is accompanied by the transformation of the anti-tumor effects and cancer-promoting effects of autophagy. Regarding the regulation of drug resistance against multi-kinase inhibitors, the diverse role of autophagy is environment-dependent. Phosphorylated p62 and Keap1 accumulate in autophagy-deficient tumors and form aggregates, leading to the continued activation of Nrf2, which eventually contributes to HCC growth. Phosphorylated p62-dependent Nrf2 activation can be abrogated by K67, which is a specific inhibitor of the interaction between phosphorylated p62 and Keap1. K67 proficiently inhibits the proliferation of HCC and diminishes the tolerance of HCC to anticancer agents (sorafenib and cisplatin), especially in patients diagnosed with HCV (53). Conversely, HCC cells sometimes compensate by accelerating autophagy flux to enhance their own growth and resist the anti-tumor effects of HGF-MET kinase-targeted drugs, such as Onartuzumab (Genentech) and Tivantinib (ArQule). Chloroquine (CQ) effectively abrogates the autophagy flux and can significantly increase the inhibitory effect of HGF-MET kinase-targeted inhibitor on the growth of HCC. These drugs when applied in combination, synergistically block the synthesis of DNA, triglyceride, and aspartate with negligible cytotoxicity (56).

#### Novel therapeutic strategies

Recent advances in cancer therapeutics have demonstrated that oncolytic Newcastle disease virus (NDV) can selectively treat tumors without affecting the noncancerous healthy cells. In addition to direct oncolysis, NDV can also activate the immune system and prompt tumor rejection by eliciting specific or non-specific anti-tumor immune responses. However, NDV infection can induce aerobic glycolysis in HCC cells thereby establishing the immunosuppressive microenvironment. To circumvent this limitation, dichloroacetate (DCA), an inhibitor of PDK, which can inhibit glycolysis in a dose-dependent manner, is used in conjunction to prevent establishing an immunosuppressive microenvironment. DCA improves the anti-tumor efficacy of NDV by enhancing the anti-tumor immune response and prolongs the survival time of ascites and subcutaneous HCC mouse models. In addition, DCA increases the replication of NDV in HCC in a PDK-1-dependent manner (112). Another promising treatment option is the use of Lauric acid. It is a 12-carbon fatty acid (C12), metabolized by hepatocytes to produce dodecanedioic acid (DDDA), a highly toxic dicarboxylic acid. In hepatoblastoma (HB) mouse models, C12 and/or DDDA diet induces extensive necrosis of tumor cells and exhibits a survival benefit compared to normal diet (22.8-28.6 weeks vs 14.2 weeks). C12 or DDDA dietary supplements provide a non-toxic, inexpensive, and possibly compatible treatment combined with standard radiotherapy (113). Research about transplanted tumors in nude mice has demonstrated that tumor growth can be inhibited by downregulating HK2 and PKM2 mediated by miR-119a. An efficient delivery system has been designed using cholesterol-modified miR-199a oligo (agomiR-199a) formulated with a lipid-based delivery reagent. This can be successfully injected into the tail vein of nude mice ensuring systemic delivery of miR-199a (114). Obviously, the development of new therapeutic strategies based on targeted metabolic reprogramming in HCC also continuously enriches the patterns and prospects of future tumor therapy.

## Conclusion and perspectives

Since the Warburg effect was proposed, the research on tumor metabolism has continued to expand and renewed the impact of rewired metabolism and its contribution to carcinogenesis. The reprogrammed metabolism is rapidly gaining attention and appearing to be more pleiotropic the more it is studied. Aerobic glycolysis was initially regarded as an adaptive response due to “irreversible injuring of respiration”. Later it was amended to an active and strategized regulation of tumor cells in response to the increased demand for biosynthesis to discard the inhibitory effect of TCA cycle-induced accumulation of NADH on glucose metabolism. The unique network of metabolic regulation in HCC provides a strong foundation for the development of targeted therapeutics. Several glycolytic enzyme inhibitors have exhibited encouraging therapeutic effects *in vivo* and *in vitro*. Moreover, the combined application of sorafenib not only circumvents the problem of drug resistance, but also confers a synergistic effect of multi-drug action. In addition, the development of novel therapeutic strategies targeting HCC metabolic reprogramming, such as T3, metformin, oncolytic viruses, and intravenous delivery systems for oncolytic miRNAs, has enriched the future methods and prospects of cancer treatment.

However, the current research on tumor metabolism is mostly limited to macromolecular nutrients such as glucose, glutamine, and lipids. Research on effects of micronutrients like vitamins, trace metals, choline and other substances on HCC progression is largely generally neglected and hence poorly understood. This aspect of cancer research still needs to be explored in depth. The ability of HCC cells to seek alternative nutrient supply pathways under conditions of glucose unavailability may be a key inducing factor triggering transformation or tumorigenesis. At the same time, the molecular heterogeneity within tumors is also worth considering. Even in the same tumor, there may be diversified outcomes, such as different gene mutations leading to differential regulation of molecular networks, regional differences in nutrient supply, localized effects of stromal and inflammatory cells, and the cell-autonomous effects regulated by mutant clone expansion. All these factors may alter the metabolic preference and flexibility of tumor cells. The focus of future cancer metabolic research should be on elucidating how to combine metabolomics and/or *in vivo* isotope perfusion with histological and molecular studies, map out different metabolic domains, and associate them with specific mutation combinations or microenvironmental influences.

## Author contributions

HX, ZH and ZW wrote the manuscript. YC, JPY and YX revised and approved the manuscript. All authors contributed to the article and approved the submitted version.

## Funding

This study was funded by Hong Kong Scholars Program (XJ2020012), National Natural Science Foundation of China (81902431), Special Project of China Postdoctoral Science Foundation (2019T120279), China Postdoctoral Science Foundation (2018M641849 and 2018M640311), Postgraduate Innovative Research Project of Harbin Medical University (YJSCX2016-21HYD), Foundation of Key Laboratory of Myocardial Ischemia, Ministry of Education (KF201810) and Chen Xiaoping Foundation for the Development of Science and Technology of Hubei Province (CXPJJH11800004-001 and CXPJJH11800004-003).

## Conflict of interest

The authors declare that the research was conducted in the absence of any commercial or financial relationships that could be construed as a potential conflict of interest.

## Publisher’s Note

All claims expressed in this article are solely those of the authors and do not necessarily represent those of their affiliated organizations, or those of the publisher, the editors and the reviewers. Any product that may be evaluated in this article, or claim that may be made by its manufacturer, is not guaranteed or endorsed by the publisher.
